# Advantages of a conservative velocity interpolation (CVI) scheme for particle‐in‐cell methods with application in geodynamic modeling

**DOI:** 10.1002/2015GC005824

**Published:** 2015-06-12

**Authors:** Hongliang Wang, Roberto Agrusta, Jeroen van Hunen

**Affiliations:** ^1^ Department of Earth SciencesDurham UniversityDurhamUK; ^2^ Department of Earth Science and EngineeringImperial College LondonLondonUK

**Keywords:** conservative interpolation, divergence free, PIC method, viscosity contrast

## Abstract

The particle‐in‐cell method is generally considered a flexible and robust method to model the geodynamic problems with chemical heterogeneity. However, velocity interpolation from grid points to particle locations is often performed without considering the divergence of the velocity field, which can lead to significant particle dispersion or clustering if those particles move through regions of strong velocity gradients. This may ultimately result in cells void of particles, which, if left untreated, may, in turn, lead to numerical inaccuracies. Here we apply a two‐dimensional conservative velocity interpolation (CVI) scheme to steady state and time‐dependent flow fields with strong velocity gradients (e.g., due to large local viscosity variation) and derive and apply the three‐dimensional equivalent. We show that the introduction of CVI significantly reduces the dispersion and clustering of particles in both steady state and time‐dependent flow problems and maintains a locally steady number of particles, without the need for ad hoc remedies such as very high initial particle densities or reseeding during the calculation. We illustrate that this method provides a significant improvement to particle distributions in common geodynamic modeling problems such as subduction zones or lithosphere‐asthenosphere boundary dynamics.

## Key Points

- Introduction of a conservative velocity interpolation (CVI) scheme
- CVI reduces particle dispersion and clustering

## 1 Introduction

Chemical heterogeneities play an important role in mantle dynamics and an accurate numerical method to treat them in geodynamic models is of prime importance [*Tackley*, 1998; *McNamara and Zhong*, 2004]. Several techniques are used to track the composition field in computational fluid dynamics, and the particle‐in‐cell method (PIC), which advects composition‐carrying particles with the ambient velocity field, is found to be a very flexible and robust method to model many geodynamical problems [*Van Keken et al*., 1997; *Tackley and King*, 2003] and is commonly used in the mantle convection community [e.g., *Van Keken et al*., 1997; *Schmeling*, 2000; *Gerya and Yuen*, 2003b; *Moresi et al*., 2003; *Tackley and King*, 2003; *Ballmer et al*., 2009].

The algorithm of the PIC method to track the composition field typically involves (1) velocity interpolation from a grid of computational nodal points (hereafter collectively referred to as the mesh) to the particle locations, (2) time‐integrated advection of the particles, and 3) interpolation of the particle information to the mesh. For most problems, a second or fourth‐order Runge‐Kutta scheme usually proves to be sufficiently accurate to advect the particles [*Gerya and Yuen*, 2003b; *Moresi et al*., 2003; *McNamara and Zhong*, 2004]. Although a commonly used bilinear or biquadratic velocity interpolation to the particles may be sufficiently accurate for many flow problems [*Van Keken et al*., 1997; *Gerya and Yuen*, 2003b; *Tackley and King*, 2003], these methods interpolate the velocity components independently, without considering the divergence of the velocity field. Such interpolation schemes might induce nonphysical clustering of the particles, depending on the flow field [*Jenny et al*., 2001; *Meyer and Jenny*, 2004]. This effect may not be significant or obvious when the velocity field is rather smooth, but an unphysical distribution of the particles can become significant if strong velocity gradients are present. Ultimately, this may result in grid cells or elements totally void of particles, which are sometimes remedied using locally very high mesh resolutions and/or particle densities, or various ad hoc solutions, such as assuming a default composition for those empty cells, or repeated reseeding with additional particles [*Poliakov and Podladchikov*, 1992; *Weinberg and Schmeling*, 1992; *Edwards and Bridson*, 2012]. As we will illustrate below, this problem can be particularly severe in case of geodynamical applications with sharp viscosity contrasts and thus strong velocity gradients, such as plate interfaces in subduction zones.

An improved velocity interpolation scheme that conserves the divergence of the flow field has been developed by *Jenny et al*. [2001] and the simplified scheme for incompressible flow (i.e., divergence free) has been demonstrated that it largely eliminates the spurious distribution of particles for 2‐D incompressible flow problem [*Meyer and Jenny*, 2004]. Other types of divergence‐free interpolations have also been proposed for specific 2‐D incompressible flow field [*Vennell and Beatson*, 2009; *McNally*, 2011], although equivalent schemes for 3‐D flow are often absent. Impressed by the simplicity of the scheme in 2‐D Cartesian coordinate system as described by *Meyer and Jenny* [2004], we test it in our code and further develop the equivalent 3‐D scheme in this study. We illustrate that the divergence‐free interpolations (i.e., conservative velocity interpolation for incompressible flow) in both 2‐D and 3‐D calculations are very successful in many geodynamical scenarios where large local viscosity contrasts are common.

## 2 Method

### 2.1 Governing Equations

To illustrate the concept of particle divergence in an incompressible, infinite Prandtl‐number flow field, the following standard nondimensional governing equations for conservation of mass, momentum, energy, and composition are solved under the Boussinesq approximation:

(1) ∇⋅u=0,

(2) $$-\nabla P+\nabla \left(\eta \left(\nabla u+\nabla u^{T}\right)\right)+\left(RaT-RbC\right)e_{z}=0,$$

(3) $$\frac{\partial T}{\partial t}+u\cdot \nabla T=\nabla^{2}T,$$

(4) $$\frac{\partial C}{\partial t}+u\cdot \nabla C=0.$$

where **u**, *P*, *η*, *T*, *C*, *t*, *Ra*, and *Rb* represent velocity, pressure, viscosity, temperature, composition, time, the thermal, and compositional Rayleigh number, respectively, and **e_z_** is the vertical unit vector positive upward.

For the steady state flow problems in section 3, analytical solutions for equations (1) and (2) are applied at the nodes at every time step. In that case, particles are passively advected through the interpolated velocity field and do not affect the flow. For the time‐dependent flow problems in section 4, the flow field is solved numerically using a Cartesian finite element code Citcom [*Moresi and Solomatov*, 1995; *Zhong et al*., 2000]. In the case of active particles, the composition field carried by the particles can have a feedback on the solution of equations (2) and (3).

Equation (4) is solved by a particle‐tracking technique, in which the particles are advected at every time step by a second‐order Runge‐Kutta scheme with interpolated velocities based on the node velocities from equations (1) and (2). The compositional value is then interpolated to the finite element integration points.

### 2.2 Velocity Interpolation Scheme

**Figure 1:**
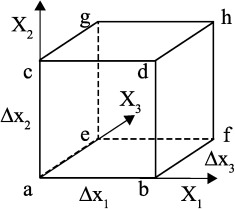
Schematic diagram to show the node name convention of a 3‐D finite element used in this study.

The nonconservative interpolation from nodal points of a local finite element to any point within the element is done using a bilinear (in 2‐D) or trilinear (3‐D) interpolation scheme with second‐order accuracy. In the general 3‐D case, this interpolated velocity *U^L^* is defined as

(5) $$\begin{array}{c} U_{i}{}^{L}\left(x_{1},x_{2},x_{3}\right)=\left(1-x_{1}\right)\left(1-x_{2}\right)\;\left[\left(1-x_{3}\right)U_{i}^{a}+x_{3}U_{i}^{e}\right]+x_{1}\;\left(1-x_{2}\right)\;\left[\left(1-x_{3}\right)U_{i}^{b}+x_{3}U_{i}^{f}\right]\\ +\left(1-x_{1}\right)x_{2}\;\left[\left(1-x_{3}\right)U_{i}^{c}+x_{3}U_{i}^{g}\right]+x_{1}\;x_{2}\;\left[\left(1-x_{3}\right)U_{i}^{d}+x_{3}U_{i}^{h}\right], \end{array}$$

where *U_i_* is the *i*th‐component of the velocity field at local coordinates (*x*_1_, *x*_2_, *x*_3_) and superscripts *a‐h* refer to the nodal points of the unit cell, as illustrated in Figure 1. The improved conservative interpolation can be derived by adding a correction term to conserve the divergence velocity field after the interpolation. The 2‐D case for the incompressible flow is provided by *Meyer and Jenny* [2004], in which correction terms are added to the bilinear interpolations (equation (5)):

(6) $$U_{i}=U_{i}{}^{L}+\Delta U_{i},$$

with Δ*U_i_* is a correction term. Here we derive the general, 3‐D version of this correction term for incompressible flow as

(7) $$\begin{array}{c} \Delta U_{1}=x_{1}\left(1-x_{1}\right)\left(C_{10}+x_{2}C_{12}\right)\\ \Delta U_{2}=x_{2}\left(1-x_{2}\right)\left(C_{20}+x_{3}C_{23}\right)\\ \Delta U_{3}=x_{3}\left(1-x_{3}\right)\left(C_{30}+x_{1}C_{31}\right), \end{array}$$

where the coefficients (*C*_10_, *C*_12_, *C*_20_, *C*_23_, *C*_30_, and *C*_31_) are defined as (see supporting information):

(8) $$\begin{array}{c} C_{12}=\frac{\Delta x_{1}}{2\Delta x_{3}}\left[-U_{3}^{a}+U_{3}^{b}+U_{3}^{c}-U_{3}^{d}+U_{3}^{e}-U_{3}^{f}-U_{3}^{g}+U_{3}^{h}\right]\\ C_{23}=\frac{\Delta x_{2}}{2\Delta x_{1}}\left[-U_{1}^{a}+U_{1}^{b}+U_{1}^{c}-U_{1}^{d}+U_{1}^{e}-U_{1}^{f}-U_{1}^{g}+U_{1}^{h}\right]\\ C_{31}=\frac{\Delta x_{3}}{2\Delta x_{2}}\left[-U_{2}^{a}+U_{2}^{b}+U_{2}^{c}-U_{2}^{d}+U_{2}^{e}-U_{2}^{f}-U_{2}^{g}+U_{2}^{h}\right]\\ C_{10}=\frac{\Delta x_{1}}{2\Delta x_{2}}\left[U_{2}^{a}-U_{2}^{c}+U_{2}^{d}-U_{2}^{b}\right]+\frac{\Delta x_{1}}{2\Delta x_{3}}\left[U_{3}^{a}-U_{3}^{e}+U_{3}^{f}-U_{3}^{b}+C_{31}\right]\\ C_{20}=\frac{\Delta x_{2}}{2\Delta x_{1}}\left[U_{1}^{a}-U_{1}^{b}+U_{1}^{d}-U_{1}^{c}+C_{12}\right]+\frac{\Delta x_{2}}{2\Delta x_{3}}\left[U_{3}^{a}-U_{3}^{e}+U_{3}^{g}-U_{3}^{c}\right]\\ C_{30}=\frac{\Delta x_{3}}{2\Delta x_{1}}\left[U_{1}^{a}-U_{1}^{b}+U_{1}^{f}-U_{1}^{e}\right]+\frac{\Delta x_{3}}{2\Delta x_{2}}\left[U_{2}^{a}-U_{2}^{c}+U_{2}^{g}-U_{2}^{e}+C_{23}\right]. \end{array}$$

In 2‐D, the interpolation and correction schemes simplify significantly by ignoring all terms associated to the third dimension (i.e., *x*_3_ = 0 and $U_{i}{}^{e}=U_{i}{}^{f}=U_{i}{}^{g}=U_{i}{}^{h}=0$), in which case equation (7) simplifies to the 2‐D incompressible scheme of *Meyer and Jenny* [2004].

Adding these corrections does not improve the order of accuracy of the interpolation (it remains a second‐order accurate scheme), but they ensure a divergence‐free velocity field over the cell.

## 3 Steady State Flow Experiments

In this section, two 2‐D steady state flow problems with analytical solutions are used to illustrate and test the nonconservative and conservative velocity interpolation, hereafter called n‐CVI (equation (6) with Δ*U_i_* = 0) and CVI (equation (6) with Δ*U_i_* ≠ 0), respectively. The flow in these problems is incompressible, so ideally, no particle convergence or divergence should occur.

### 3.1 Couette Flow

**Figure 2:**
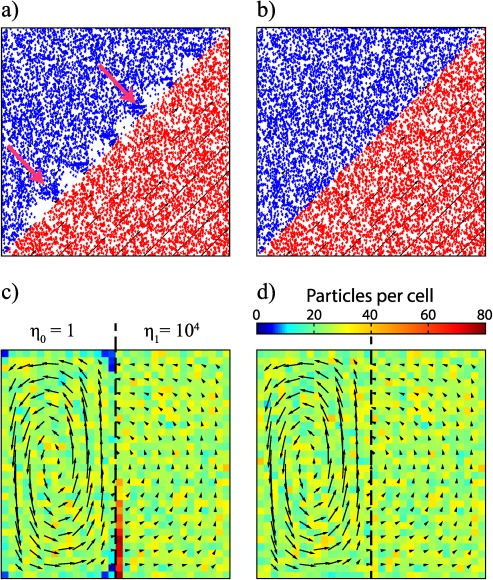
Particle distribution for the two steady state cases after 5000 time steps, with black arrows showing the velocity field at the nodes. Particle distributions obtained by a diagonal Couette flow, with red and blue areas indicating low and high viscosity, respectively. (a) The velocity is interpolated with the n‐CVI scheme. The dispersion (white areas) and clustering (highlight by pink arrows) of particles at the boundary between the two rheological layers is clearly visible. (b) The interpolation is carried out with the CVI, in which case the particle clustering is absent. SolCx test (c) with the n‐CVI scheme and (d) with the CVI scheme. The color scale indicates the number of particles per cell. Using the n‐CVI produces almost empty cells and clustering of particles at the edge of the viscosity interface, while with the CVI any clustering is virtually absent.

The first test is a simple laminar flow of viscous fluids between two relatively moving parallel plates, known as Couette flow. This flow is characterized with a constant shear stress throughout the flow domain, so analytical solutions for different viscosity layering are easily derived. We imposed the analytical solution of the velocity field for a Couette flow with two different viscous fluids (viscosity ratio of 10^3^) in a unit model domain. The flow is at a 45° angle to the boundaries of the domain, as shown in Figures 2a and 2b. To clearly illustrate the potential problem with n‐CVI scheme, a very course mesh of 8 × 8 cells is used and an initially randomly distributed set of 10^4^ particles. To provide a continuous solution in time, particles are allowed to flow into and out of the model domain at every time step.

When the n‐CVI is used, a significant pattern develops in the particle distribution, since the sharply contrasting velocities of the four corners of any cell that lies across the two viscosity domains will result in spurious velocities inside the cell due to the imperfect interpolation (Figure 2a). The particle dispersion and clustering in those cells occurs because particles in the upper part of the cell move with a much faster velocity than they should, due to the bilinear interpolation from the high velocity of the lower right node, while they will stay almost stagnant once they move to the next cell that does not contain any high‐velocity node. Figure 2b illustrates that these spurious particle distribution patterns disappear when the CVI scheme is applied.

### 3.2 solCx With Viscosity Jump of 10^4^

The second test is the analytical solution for 2‐D incompressible Stokes flow with a sharp lateral viscosity jump, developed by *Zhong* [1996], which was later termed “solCx” [*Duretz et al*., 2011]. Here we use a viscosity jump of 10^4^ in the middle of the box. The computational domain has a unit aspect ratio, and it is discretized by 32 × 32 cells. The flow is driven by an internal sinusoidal force [*Duretz et al*., 2011], with free‐slip mechanical boundary conditions. The analytical solution has been used as benchmark for high viscosity contrast experiments [*Moresi et al*., 1996; *Duretz et al*., 2011; *Thielmann et al*., 2014]. The source code to calculate the analytical solution is provided as part of the open source software Underworld [*Moresi et al*., 2007].

A set of 25,600 initially randomly distributed particles (∼25 per cell) is advected using the analytical velocity solution at the nodes and interpolated from the nodes to the particles for every time step. As shown in Figure 2c, particle clustering forms with the n‐CVI within 5000 time steps advection. Particle clustering develops near the viscosity jump where the strong velocity gradient is located. Strong gradients in the velocity field (which is originally divergence free at the cell nodes) for cells that cross the viscosity interface result in an interpolated velocity that is not divergence free anymore, because the interpolation scheme does not explicit conserve the divergence (Figure 2c). For the CVI scheme, the interpolation is explicitly divergence free, and therefore particles do not cluster (Figure 2d).

## 4 Time‐Dependent Flow in Geodynamical Applications

To illustrate the advantage of the CVI scheme for more geodynamically interesting problems, three time‐dependent flow problems in which particles affect the flow field are modeled with different particle velocity interpolations in this section. We first compare our results with the standard benchmark problem from *van Keken et al*. [1997], and then we test two types of specific geodynamic problems.

### 4.1 Rayleigh‐Taylor Instability With a Viscosity Contrast

**Figure 3:**
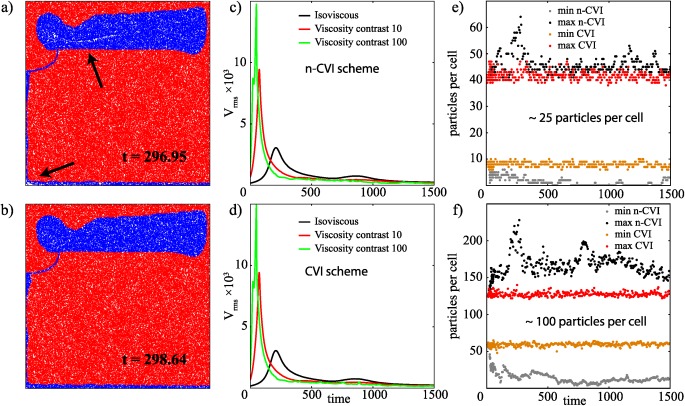
Rayleigh‐Taylor instability benchmark test after *Van Keken et al*. [1997]. Particle distribution for the case with a viscosity contrast of 100 (red and blue are high and low viscosity, respectively) obtained using the (a) n‐CVI and (b) CVI scheme, respectively. The black arrows in Figure 3a indicate gaps in the particle distribution. Time series of the velocity rms for three viscosity contrasts (Δ*η* = 1, 10, 100) for the (c) n‐CVI and (d) CVI scheme, respectively. The maximum and minimum number of particles per cell across all cells for Δ*η* = 100 calculations for an initial particle density of (e) ∼25 particle per cell and (f) ∼100 particles per cell.

We use a Rayleigh‐Taylor instability [*van Keken et al*., 1997] to test the bilinear interpolation for a thermochemical convection problem. This benchmark case has become a rather standard test in the geodynamical community for particle‐based methods. The convection is driven by compositional density differences. The composition is advected with particles, and for the cases with viscosity contrast between the layers, the viscosity is also carried by the particles. So an important difference with the previous, analytical test cases is that particles actively affect the flow field. Figures 3a and 3b show flow results obtained for the case with viscosity contrast of 100 performed in a model domain discretized by 64 × 64 cells and filled with ∼25 particles per cell. Figure 3a illustrates the gaps in the particle distribution using the n‐CVI, whereas with the CVI scheme, the particles remain proportionally distributed throughout the domain (Figure 3b). The interpolation method does not significantly affect the flow pattern, as shown by the time series for the root‐mean‐square velocity of the three cases demonstrated (Figures 3c and 3d). The advantage of using the CVI scheme is more clearly illustrated in Figures 3e and 3f, where the maximum and the minimum number of particles per cell throughout the simulation are plotted. This shows how, in the n‐CVI scheme, the number of particles per cell is strongly time dependent, with some elements eventually being completely void of particles, whereas the CVI scheme maintains a statistically constant particle density everywhere.

### 4.2 2‐D Subduction Dynamics

**Figure 4:**
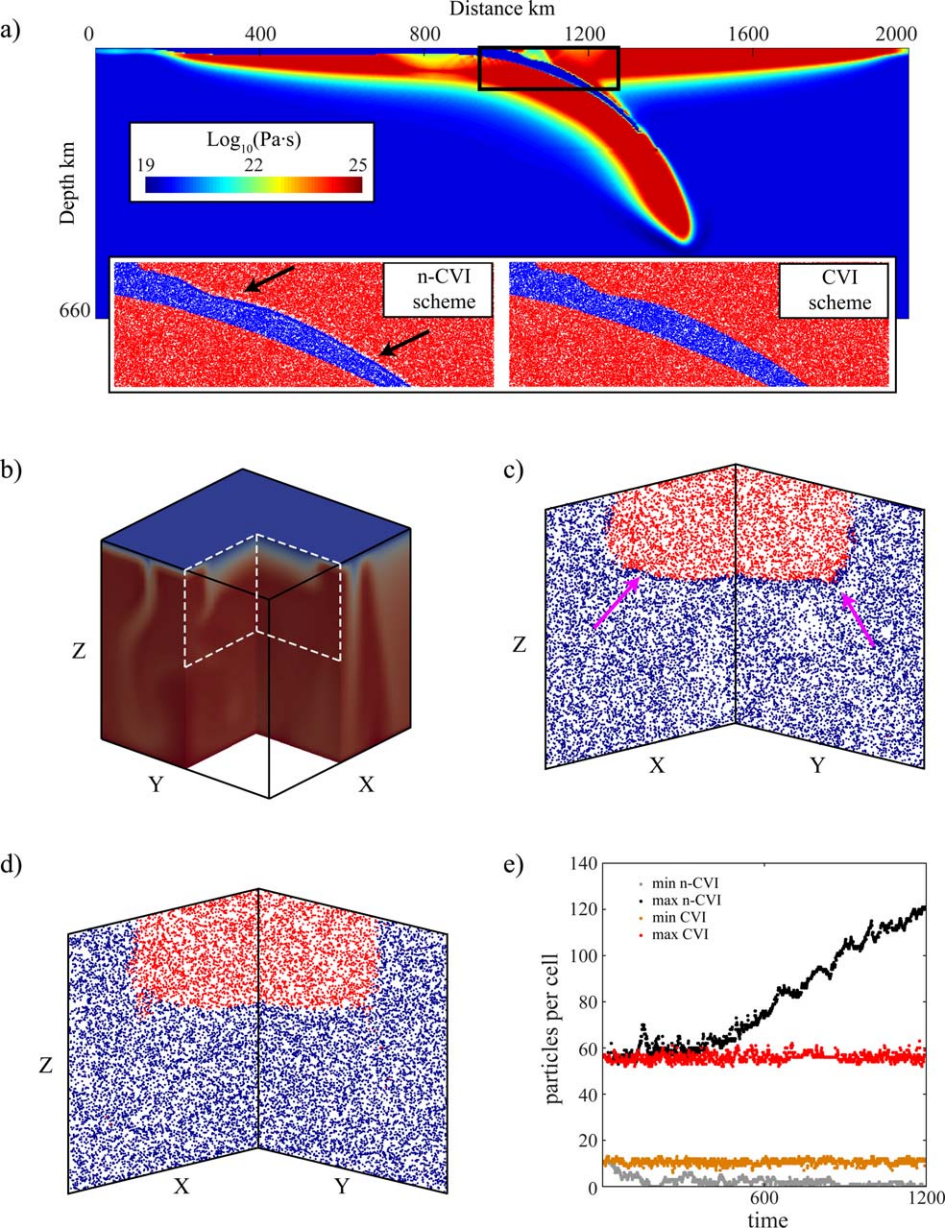
Two examples on the effect of the different velocity interpolation schemes for geodynamically relevant scenarios in 2‐D and 3‐D. (a) Particle distribution around a weak layer that decouples converging plates in a subduction zone. (bottom left inset) Particle clustering develops at the interface between the weak layer and the overriding plate by using the n‐CVI scheme, whereas (bottom right inset) particles remain evenly distributed by using the CVI scheme. (b) Temperature field near an idealized buoyant craton in a 3‐D scenario, with particle distributions obtained with the (c) n‐CVI and (d) CVI scheme. The arrows point toward occurrences of particle clustering. (e) Time series of the maximum and minimum number of particles per element for each of the two interpolation schemes in the 3‐D craton model.

Converging plates in subduction zones are decoupled by a thin weak layer [e.g., *Agrusta et al*., 2014] (Figure 4a), and here we explore how intense shearing and local large viscosity contrasts affect the velocity interpolation for each of the interpolation schemes. The dynamics are calculated using a finite element method, in which a ∼15 km thick, weak (10^20^ Pa s) decoupling layer (down to 200 km depth) between the converging plates is modeled using active particles. The model domain represents the upper mantle, with a box height of 660 km and an aspect ratio of 3, discretized into 520 × 127 elements. The ∼3 × 10^6^ particles are distributed initially randomly across the domain. The box resolution is refined vertically and horizontally to better resolve the weak layer and area at subduction trench. For a detailed description of the numerical setup and rheological model, see *Agrusta et al*. [2014].

A model snapshot at 2.3 Myr (Figure 4a) illustrates how the sharp viscosity contrast between the rigid plate and the weak layer generates a high velocity gradient that induces the particles to accumulate in the weak layer and leaves voids above it with the n‐CVI scheme (Figure 4a, left), whereas the CVI scheme prevents this behavior (Figure 4a, right). This particle behavior is very similar to the Couette flow illustrated above.

### 4.3 3‐D Lithosphere Dynamics

Another example of a geodynamical scenario in which particle distribution can be significantly affected is the long‐term interaction between the base of the lithosphere and the convecting mantle. Here we test the CVI scheme on a 3‐D model of a very viscous cratonic root in a much weaker thermochemically convecting mantle that has often been studied in 2‐D situations [*Lenardic et al*., 2003; *O'Neill et al*., 2008; *Wang et al*., 2014, 2015]. The computational domain is 660 km deep with a unit aspect ratio. To represent a buoyant craton, a half‐sphere, compositionally different from the surrounding mantle is situated at the top of the cube (Figures 4b–4e). The viscosity contrast between the half‐sphere cratonic root and the mantle is 10^3^. The initial internal temperature field is 1350°C everywhere. *T* = 0 and *T* = 1350°C are imposed on the surface and bottom, respectively, with zero heat flux on the sides and with free‐slip mechanical boundary conditions everywhere. We use a coarse mesh resolution of 33 × 33 × 33 cells with 10^6^ particles. Figure 4b shows the temperature field after a dimensionless time of 1200. The particle distribution in cross‐section slices of one cell width, projected in a cross section, shows the particle distribution with the n‐CVI and CVI schemes in Figures 4c and 4d, respectively. Similar to the 2‐D calculations, the trilinear n‐CVI induces particle clustering near the compositional boundary (Figure 4c), whereas the 3‐D CVI scheme maintains a homogenous particle distribution (Figure 4d). With the n‐CVI, the minimum particle number reaches zero quickly and remains small afterward; the maximum particle number keeps increasing steadily, which illustrates the ongoing clustering of particles. In contrast, the minimum and maximum particle count per cell stays between 10 and 55 with the CVI scheme, which illustrates again a persistently homogenous particle distribution through time (Figure 4e).

## 5 Discussion and Conclusion

In this study, we reported a solution for a commonly observed problem in geodynamic modeling related to the PIC method. Using the analytical solution of two steady state flow problems, we demonstrate that the commonly observed clustering and dispersion of particles in a PIC method is insensitive to numerical discretization techniques or particle advection methods, but instead is caused by the nonconservative interpolation method of the velocity field from the cell nodes to the particles. The conservative velocity interpolation (CVI) proposed in this study solves this problem for incompressible flow by ensuring a divergence‐free velocity field for the particles. We illustrate that this method works very well for both steady state flow problems and geodynamically more complex and relevant time‐dependent flow problems.

### 5.1 Numerical Advantages and Wider Applicability

Maintaining a certain minimum particle density in every computation cell is important for the success of PIC methods [*van Keken et al*., 1997; *Tackley and King*, 2003]. However, significant clustering and dispersion of particles commonly occurs in the presence of strong velocity gradients, e.g., due to locally high viscosity contrasts. Using a very high particle density might help to avoid significant gaps in the particle distribution but not always, and such remedy quickly becomes computationally expensive, especially in 3‐D models. Reseeding particles to the areas approaching low particle density may appear to solve the problem but does not have solid physical base. Our results show that how the particle distribution problem can easily be solved by using a conservative velocity interpolation (i.e., divergence‐free interpolation for incompressible flow) from cell nodes to particles. This method has the advantage that the divergence property of the velocity field is maintained [*Jenny et al*., 2001; *Meyer and Jenny*, 2004], which is physically reasonable and requires negligibly higher computational costs.

The presented new interpolation scheme has been applied to four‐node quadrilateral cells in a divergence‐free flow field (i.e., incompressible flow), for which the proposed solution is very easily implemented. But the advantages of a conservative interpolation could apply more generally to compressible flow problems [*Jenny et al*., 2001] and more complex meshing techniques. However, some modifications are required to apply this scheme to more complex meshing techniques. For example, staggered grids are commonly used in finite difference and finite volume methods [e.g., *Gerya and Yuen*, 2003b] and have different node locations for the different velocity components. Therefore, an adapted implementation of the CVI scheme is required for more complex grid configurations, which will be the topic of future investigation.

### 5.2 Geodynamic Applications

With the arrival of new numerical techniques and increased computational capacity, the geodynamical modeling of chemical heterogeneity has become more and more prominent [e.g., *Gerya and Yuen*, 2003a; *McNamara and Zhong*, 2004; *Tackley*, 2008]. Examples include varying mineral compositions, or volatile content, which can have a substantial effect on the rheology of the crust and mantle [*Hirth and Kohlstedt*, 1996; *Karato*, 2006; *Keefner et al*., 2011]. These slow or nondiffusive properties can create and maintain sharp local viscosity contrasts and strong velocity gradients. Here we show how, in such scenarios, nonconservative velocity interpolation such as the bilinear/trilinear scheme could lead to significant clustering and dispersion of particles. The 2‐D and 3‐D conservative velocity interpolation (CVI) schemes presented in this study provide a simple, effective way to improve the PIC method for the incompressible flow problems under this situation. CVI is a physically correct interpolation scheme that is easily implemented and maintains statistically constant particle densities, thereby avoiding particle dispersion and locally decreasing particle densities over time. Initial particle densities can therefore be relatively low, which improves the computation efficiency, especially for 3‐D calculations.

## Supporting information

Supporting Information S1
