# Supplementary material for: Advantages of a conservative velocity interpolation (CVI) scheme for particle‐in‐cell methods with application in geodynamic modeling
Source: Geochem Geophys Geosyst. 2015 Jun 12;16(6):2015–23. doi: 10.1002/2015GC005824 (PMC5089062; doi:10.1002/2015GC005824)
Supplement: Supplementary file 1 — Supporting Information S1 [file GGGE-16-2015-s001.docx]

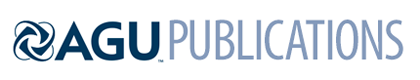


*Geochemistry, Geophysics, Geosystems*

Supporting Information for

**Advantages of a conservative velocity interpolation (CVI) scheme for particle-in-cell methods with application in geodynamic modelling**

Hongliang Wang^1^, Roberto Agrusta^1,2^, Jeroen van Hunen^1^

^1^Department of Earth Sciences, Durham University, Durham, UK

^2^Department of Earth Science and Engineering, Imperial College London, London, UK

Corresponding author: hongliang.wang@durham.ac.uk

**Contents of this file**

Text S1

**Introduction**

This supporting material contains the derivation process of the three-dimensional (3D) conservative velocity interpolation (CVI) for incompressible flow problem. We extended the two-dimensional (2D) conservative velocity interpolation in *Meyer and Jenny* [2004] based on the idea of adding correction items to the tranditional interpolations. The presented method and result only apply to 4 nodes normal grid but not the staggered grid in which the velocity points are not collocated.

Text S1

We focus on the incompressible flow problem, in which the conservative velocity interpolation is actually divergence-free interpolation. Here we first describe the 2D divergence-free velocity interpolation by *Meyer and Jenny* [ 2004] and further derive 3D divergence-free interpolation formulations.

In a 4-node 2D rectangular cell system, bilinear interpolation provides a simple and quick interpolation scheme and is widely used. If we transform the rectangular cells into unit squares (Fig 1), the interpolation we used can be written as:

${U_{i}}^{L}\left( x_{1,}x_{2} \right)=\left\{ \left( 1-x_{1} \right)\left( 1-x_{2} \right), x_{1}\left( 1-x_{2} \right), \left( 1-x_{1} \right)x_{2}, x_{1}x_{2} \right\}\cdot{\{U}_{i}^{a}, U_{i}^{b}, U_{i}^{c},U_{i}^{d}$} , (1)

where the two velocity components are interpolated independently as two separate scalars without considering the divergence of the vector field need to be 0.

$\frac{\partial U_{1}}{{\partial x}_{1}}+\frac{\partial U_{2}}{{\partial x}_{2}}=0$ (2)

The 2D divergence-free interpolation is achieved by adding correction items as follows [*Meyer and Jenny*, 2004]:

$U_{i}=U_{i}^{L}+ \Delta U_{i}$, (3)

$\Delta U_{1}=\frac{\Delta x_{1}}{2\Delta x_{2}}x_{1}\left( 1-x_{1} \right)\left( U_{2}^{a}-U_{2}^{b}-U_{2}^{c}+U_{2}^{d} \right)$, (4)

$\Delta U_{2}=\frac{\Delta x_{2}}{2\Delta x_{1}}x_{2}(1-x_{2})(U_{1}^{a}-U_{1}^{b}-U_{1}^{c}+U_{1}^{d})$. (5)

In Eqs (4) and (5), the correction item for each velocity component is calculated based on the other velocity component of the nodes. We extend this approach into 3D situation by adding a quadratic item of x_i_ to each velocity component:

$\begin{matrix} U_{i}^{L}\left(x_{1},x_{2},x_{3}\right)=\left(1-x_{1}\right)\left(1-x_{2}\right) \left[\left(1-x_{3}\right)U_{i}^{a}+x_{3}U_{i}^{e}\right]+ \\ x_{1} \left(1-x_{2}\right) \left[\left(1-x_{3}\right)U_{i}^{b}+x_{3}U_{i}^{f}\right]+ \\ \left(1-x_{1}\right) x_{2} \left[\left(1-x_{3}\right)U_{i}^{c}+x_{3}U_{i}^{g}\right]+ \\ x_{1} x_{2} \left[\left(1-x_{3}\right)U_{i}^{d}+x_{3}U_{i}^{h}\right] \end{matrix}$, (6)

$\Delta U_{1}=x_{1}\left(1-x_{1}\right)\left(C_{10}+x_{2}C_{12}\right)$, (7)

$\Delta U_{2}=x_{2}\left(1-x_{2}\right)\left(C_{20}+x_{3}C_{23}\right)$ , (8)

$\Delta U_{3}=x_{3}\left(1-x_{3}\right)\left(C_{30}+x_{1}C_{31}\right)$. (9)

The coefficients of these item (C_10_, C_12_, C_20_, C_23_, C_30_, C_31_) in Eqs (7-9) is to be determined. They should satisfy the following divergence free condition for 3D incompressible flow field:

$\frac{\partial U_{1}}{\partial x_{1}}+\frac{\partial U_{2}}{\partial x_{2}}+\frac{\partial U_{3}}{\partial x_{3}}=0$ . (10)

Thus, we take the first derivatives of U_i_ with respect to x_i_  based on Eqs (3, 6,7,8,9):

$$\Delta x_{1}\frac{\partial U_{1}}{\partial x_{1}}=\left( 1-x_{2} \right)*\left( 1-x_{3} \right)*\left[ U_{1}^{b}-U_{1}^{a} \right]+x_{2}*\left( 1-x_{3} \right)*\left[ U_{1}^{d}-U_{1}^{c} \right]$$

$+\left( 1-x_{2} \right)*x_{3}*\left( U_{1}^{f}-U_{1}^{e} \right)+x_{2}*x_{3}*\left( U_{1}^{h}-U_{1}^{g} \right)$

$+(1-{2x}_{1})*(C_{10}+C_{12}*x_{2})$ (11)

$$\Delta x_{2}\frac{\partial U_{2}}{\partial x_{2}}=\left( 1-x_{1} \right)*\left( 1-x_{3} \right)*\left[ U_{2}^{c}-U_{2}^{a} \right]+x_{1}*\left( 1-x_{3} \right)*\left[ U_{2}^{d}-U_{2}^{b} \right]$$

$+\left( 1-x_{1} \right)*x_{3}*\left( U_{2}^{g}-U_{2}^{e} \right)+x_{1}*x_{3}*\left( U_{2}^{h}-U_{2}^{f} \right)$

$+(1-{2x}_{2})*(C_{20}+C_{23}*x_{3})$ (12)

$$\Delta x_{3}\frac{\partial U_{3}}{\partial x_{3}}=\left( 1-x_{1} \right)*\left( 1-x_{2} \right)*\left[ U_{3}^{e}-U_{3}^{a} \right]+x_{1}*\left( 1-x_{2} \right)*\left[ U_{3}^{f}-U_{3}^{b} \right]$$

$+\left( 1-x_{1} \right)*x_{2}*\left( U_{3}^{g}-U_{3}^{c} \right)+x_{1}*x_{2}*\left( U_{3}^{h}-U_{3}^{d} \right)$

$+(1-{2x}_{3})*(C_{30}+C_{31}*x_{1})$ (13)

Substitute Eqs (11-13) in to Eq (10) and we have an identical equation with the six unknowns (C_10_, C_12_, C_20_, C_23_, C_30_, C_31_). As the result, the following items should have their coefficients to be zeros: 1, x_1_, x_2_, x_3_, x_1_x_2_, x_2_x_3_, x_3_x_1_, which lead to 7 equations:

1 : $\frac{1}{\Delta x_{1}}[U_{1}^{b}-U_{1}^{a}+C_{10}]+\frac{1}{\Delta x_{2}}[U_{2}^{c}-U_{2}^{a}+C_{20}]+\frac{1}{\Delta x_{3}}[U_{3}^{e}-U_{3}^{a}+C_{30}]=0$

x_1_: $\frac{1}{\Delta x_{1}}[-{2C}_{10}]+\frac{1}{\Delta x_{2}}[U_{2}^{a}-U_{2}^{c}+U_{2}^{d}-U_{2}^{b}]+\frac{1}{\Delta x_{3}}[U_{3}^{a}-U_{3}^{e}+U_{3}^{f}-U_{3}^{b}+C_{31}]=0$

x_2_ : $\frac{1}{\Delta x_{1}}[U_{1}^{a}-U_{1}^{b}+U_{1}^{d}-U_{1}^{c}+C_{12}]+\frac{1}{\Delta x_{2}}[-{2C}_{20}]+\frac{1}{\Delta x_{3}}[U_{3}^{a}-U_{3}^{e}+U_{3}^{g}-U_{3}^{c}]=0$

x_3_ : $\frac{1}{\Delta x_{1}}[U_{1}^{a}-U_{1}^{b}+U_{1}^{f}-U_{1}^{e}]+\frac{1}{\Delta x_{2}}[U_{2}^{a}-U_{2}^{c}+U_{2}^{g}-U_{2}^{e}+C_{23}]+\frac{1}{\Delta x_{3}}[-{2C}_{30}]=0$

x_1_x_2_: $\frac{1}{\Delta x_{1}}[-{2C}_{12}]+\frac{1}{\Delta x_{3}}[U_{3}^{e}-U_{3}^{a}+U_{3}^{b}-U_{3}^{f}+U_{3}^{c}-U_{3}^{g}+U_{3}^{h}-U_{3}^{d}]=0$

x_2_x_3_: $\frac{1}{\Delta x_{1}}[U_{1}^{b}-U_{1}^{a}+U_{1}^{c}-U_{1}^{d}+U_{1}^{e}-U_{1}^{f}+U_{1}^{h}-U_{1}^{g}]+\frac{1}{\Delta x_{2}}[-{2C}_{23}]=0$

x_3_x_1_: $\frac{1}{\Delta x_{2}}[U_{2}^{c}-U_{2}^{a}+U_{2}^{b}-U_{2}^{d}+U_{2}^{e}-U_{2}^{g}+U_{2}^{h}-U_{2}^{f}]+\frac{1}{\Delta x_{3}}[-{2C}_{31}]=0$

From (10), we could also have

$\begin{aligned} \frac{(U_{1}^{a}+U_{1}^{c}+U_{1}^{e}+U_{1}^{g}-U_{1}^{b}-U_{1}^{d}-U_{1}^{f}-U_{1}^{h})}{\Delta x1} \\ \frac{+(U_{2}^{a}+U_{2}^{b}+U_{2}^{e}+U_{2}^{f}-U_{2}^{c}-U_{2}^{d}-U_{2}^{g}-U_{2}^{h})}{\Delta x2} \\ \frac{+(U_{3}^{a}+U_{3}^{b}+U_{3}^{c}+U_{3}^{d}-U_{3}^{e}-U_{3}^{f}-U_{3}^{g}-U_{3}^{h})}{\Delta x3}=0 \end{aligned}$ (14)

which reduces 7 equations into 6 independent equations. Therefore, the coefficients in Eqs (7-9) as the six unknowns are determined as follows :

$C_{12}=\frac{\Delta x_{1}}{2\Delta x_{3}}[U_{3}^{e}-U_{3}^{a}+U_{3}^{b}-U_{3}^{f}+U_{3}^{c}-U_{3}^{g}+U_{3}^{h}-U_{3}^{d}]$,

$C_{23}=\frac{\Delta x_{2}}{2\Delta x_{1}}[U_{1}^{b}-U_{1}^{a}+U_{1}^{c}-U_{1}^{d}+U_{1}^{e}-U_{1}^{f}+U_{1}^{h}-U_{1}^{g}]$,

$C_{31}=\frac{\Delta x3}{2\Delta x_{2}}[U_{2}^{c}-U_{2}^{a}+U_{2}^{b}-U_{2}^{d}+U_{2}^{e}-U_{2}^{g}+U_{2}^{h}-U_{2}^{f}]$,

$C_{10}=\frac{\Delta x_{1}}{2\Delta x_{2}}[U_{2}^{a}-U_{2}^{c}+U_{2}^{d}-U_{2}^{b}]+\frac{\Delta x_{1}}{2\Delta x_{3}}[U_{3}^{a}-U_{3}^{e}+U_{3}^{f}-U_{3}^{b}+C_{31}]$,

$C_{20}=\frac{\Delta x_{2}}{2\Delta x_{1}}[U_{1}^{a}-U_{1}^{b}+U_{1}^{d}-U_{1}^{c}+C_{12}]+\frac{\Delta x_{2}}{2\Delta x_{3}}[U_{3}^{a}-U_{3}^{e}+U_{3}^{g}-U_{3}^{c}]$,

$C_{30}=\frac{\Delta x_{3}}{2\Delta x_{1}}[U_{1}^{a}-U_{1}^{b}+U_{1}^{f}-U_{1}^{e}]+\frac{\Delta x_{3}}{2\Delta x_{2}}[U_{2}^{a}-U_{2}^{c}+U_{2}^{g}-U_{2}^{e}+C_{23}]$.

**Reference**

Meyer, D., and P. Jenny (2004), Conservative velocity interpolation for PDF methods, PAMM, 467, 466–467, doi:10.1002/pamm.200410.
